# Neurochemical changes in basal ganglia affect time perception in parkinsonians

**DOI:** 10.1186/s12929-018-0428-2

**Published:** 2018-03-19

**Authors:** Francisco Magalhães, Kaline Rocha, Victor Marinho, Jéssica Ribeiro, Thomaz Oliveira, Carla Ayres, Thalys Bento, Francisca Leite, Daya Gupta, Victor Hugo Bastos, Bruna Velasques, Pedro Ribeiro, Marco Orsini, Silmar Teixeira

**Affiliations:** 1 Brain Mapping and Plasticity Laboratory, Federal University of Piauí, Av. São Sebastião n° 2819, Nossa Sra. de Fátima, Parnaíba, PI 64202-020 Brazil; 2 The Northeast Biotechnology Network (RENORBIO), Federal University of Piauí, Teresina, Brazil; 3 Department of Biology, Camden County College, Blackwood, NJ USA; 4 Laboratory of Brain Mapping and Functionality, Federal University of Piauí, Parnaíba, Brazil; 5 Brain Mapping and Sensory-Motor Integration Laboratory, Psychiatry Institute of Federal University of Rio de Janeiro, Rio de Janeiro, Brazil; 6 Rehabilitation Science Program, Analysis of Human Movement Laboratory, Augusto Motta University Center, Rio de Janeiro, Brazil; 7 Program Professional Master in Applied Science in Health/UNISUAM, Av. Paris, 84, Bonsucesso, Rio de Janeiro, RJ 21041-020 Brazil; 8 Brain Mapping and Sensory Motor Integration Laboratory, Institute of Psychiatry of Federal University of Rio de Janeiro, Av. Venceslau Braz, 71 - Botafogo, Rio de Janeiro, RJ 22290-140 Brazil

**Keywords:** Parkinson’s disease, Time perception, Dopamine, Basal ganglia

## Abstract

**Background:**

Parkinson’s disease is described as resulting from dopaminergic cells progressive degeneration, specifically in the substantia nigra pars compacta that influence the voluntary movements control, decision making and time perception.

**Aim:**

This review had a goal to update the relation between time perception and Parkinson’s Disease.

**Methodology:**

We used the PRISMA methodology for this investigation built guided for subjects dopaminergic dysfunction in the time judgment, pharmacological models with levodopa and new studies on the time perception in Parkinson’s Disease. We researched on databases Scielo, Pubmed / Medline and ISI Web of Knowledge on August 2017 and repeated in September 2017 and February 2018 using terms and associations relevant for obtaining articles in English about the aspects neurobiology incorporated in time perception. No publication status or restriction of publication date was imposed, but we used as exclusion criteria: dissertations, book reviews, conferences or editorial work.

**Results/Discussion:**

We have demonstrated that the time cognitive processes are underlying to performance in cognitive tasks and that many are the brain areas and functions involved and the modulators in the time perception performance.

**Conclusions:**

The influence of dopaminergic on Parkinson’s Disease is an important research tool in Neuroscience while allowing for the search for clarifications regarding behavioral phenotypes of Parkinson’s disease patients and to study the areas of the brain that are involved in the dopaminergic circuit and their integration with the time perception mechanisms.

## Background

Parkinson’s disease (PD) is characterized as resulting from dopaminergic cells progressive degeneration in the midbrain, specifically in the substantia nigra pars compacta that influence the voluntary movements control and decision making [1, 2]. Thus, dopamine chronic reduction contributes to cognitive and motor changes inbuilt in the time perception (TP) [3–10], especially in the range of the supra-seconds [6, 11–14].

A well-known hypothesis in the timing research is that temporal processing in the milliseconds to seconds range involves the basal ganglia and is modulated by the dopaminergic level. The dopamine is associated with the internal clock speed, which is consistent with its effect on the internal pacemaker rate that varies between individuals, leading to a ‘faster’ clock for some and ‘slower’ clock for others. Supporting the basal ganglia role in time perception, PD patients usually have severe deficits on various temporal ranges [3–5]. However, the exact nature of PD timing problems is still elusive. The basal ganglia involvement and the dopamine level in timing judgment would thus explain the PD participants’ deficit in the most commonly used cognitive tasks (e.g. time estimation, time reproduction, and time production) [15–17]. For example, Feher et al. [18] observed timing tasks performance in young, healthy and elderly individuals with PD. The results showed that PD patients present greater inaccuracy in TP and decision-making. However, some studies reported that the temporal deficit associated with PD might be explained by impairment of other cognitive and motor processes, as a means of elucidating the physiology of the internal clock, through combinations of tools, such as genetic markers related to time perception, neuroimaging and psychophysical evaluations [19].

This review has a goal to update the relation between the time intervals interpretation and PD. Including time perception neurobiological aspects (e.g. memory, motor control) in PD, dopaminergic dysfunction in the timing judgment, pharmacological models using levodopa, and new studies on the time perception in parkinsonians. Therefore, we systematically evaluate how neurobiological deficits in neurotransmission modulate perceptual mechanisms, since it modifies the neural activity proportion in essential brain areas and structures in timing (e.g. prefrontal cortex, parietal cortex, and basal ganglia) [9, 13]. Our study shows that deficits in neural inputs during timing tasks in PD patients promote differences in the timing judgment performance, and thus, becomes a relevant factor for neuroscience in the search to elucidate pathological endophenotypes associated with neural synchronism for encoding sub seconds and supra seconds [8–11].

## The present study

We conducted a study that demonstrates the time perception neurobiological aspects (memory, motor control) in PD, in dopaminergic dysfunction through genetic and pharmacological paradigms, as well as new studies on the time perception in PD. Initially, we sought to demonstrate the central nervous system (CNS) functioning in time perception activities, as well as neuroanatomic, neurochemical and genetic relations. Time perception is a necessary capacity for the cognitive and motor tasks performance, which is deficient in PD [20, 21]. Accordingly, our findings provide an updated picture for PD and TP, pointing out the art state, limitations and future directions for research on the topic.

## Methodology

The systematic review was conducted in line with the Preferred Reporting Items for Systematic Reviews and Meta-Analyses (PRISMA statement). This study consists of a review of English language research articles about the following: Neurobiological aspects inbuilt in the TP (i.e. memory and motor control), dopaminergic dysfunction in the timing judgment, pharmacological models with levodopa and new studies on timing in PD. We included in our study: reviews, meta-analysis, case reports and original papers. No publication status or publication date restriction was imposed. We used as exclusion criteria: dissertations, book reviews, conference or editorial work. The results were analyzed, and papers deemed to be relevant and with acceptable quality were included in the analysis.

### Information sources

The online searches in the Scielo, Pubmed/Medline and ISI Web of Knowledge (1989-present) databases were initially performed in August 2017 and repeated in September 2017 and February 2018 using relevant terms: [Neurophysiology and time perception], [dopamine and timing], [Parkinson’s disease and time perception], [cognitive aspects and time perception], [motor control and time perception], [Levodopa and time perception], [memory and time perception]. In two moments, the first one was aimed at neuroanatomical, neuropharmacological and genetic aspects that could allow the time intervals interpretation in PD patients, whereas in the second moment we focused on memory, motor performance and new studies related to time perception in PD patients. Abstracts were examined to the research question, and if the study appeared relevant, then the full text was retrieved. Reference lists of identified articles were searched for additional studies.

### Study selection

The chosen studies had met the following inclusion criteria: Initially, retrieved papers from each database were compared to remove duplicate records. Papers were then screened for eligibility based on the title and abstract, and if necessary the full-text publication was reviewed. Types of neurobiological modalities: Studies were included if they investigated the structural connectivity between TP and cognitive aspects. Functional connectivity studies based on neurotransmission with genetic and pharmacological aspects applied to time were also included.

### In summary

#### Study design

Case reports, meta-analysis, original papers and reviews were included.

#### Population

A population study composed of healthy and/or PD individuals (young adults, middle-aged and elderly). In addition, ‘organism models’ using rats.

#### Intervention

Neurobiological interventions were defined as any intervention with the capacity to influence the TP mediated by cerebral neurochemistry (tasks: time estimation, time reproduction, verbal estimation, motor reproduction, temporal discrimination).

#### Results

The primary outcome measure was the change in the TP using a timing task with visual or auditory stimuli, neurochemical and genetic factors that modify neural synchronism in cognition, as well as PD phenotypes that distort the TP.

**Fig. 1:**
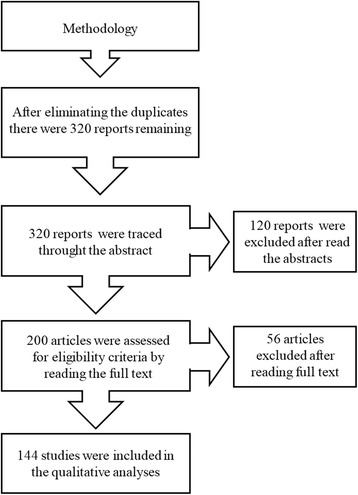
Procedure for systematic review

The database search identified 320 unique publications for study descriptors combinations. These documents were assessed for eligibility based on title and abstract; 200 were classified as meeting the eligibility criteria. The next step in the process involved full-text screening of potentially relevant articles; these were subsequently considered as fulfilling the eligibility criteria. Thus, in total, 144 studies were included in the review (Fig. 1).

### Study selection and data extraction

Four reviewers (FM, VM, KR, and JR) independently read the titles and/or abstracts of the identified papers and eliminated irrelevant studies. Studies considered eligible for inclusion were read in full and their suitability for inclusion was determined independently by four reviewers (FM, VM, KR, and JR). Disagreements were managed by consensus. However, if this was not successful, the consensus was sought by a fifth reviewer (ST).

Data were extracted based on study design and setting. Some authors were contacted to provide supplementary information when insufficient data were provided in the study. The authors of two studies were contacted for further information having read their titles and abstracts.

## Results

Regarding the neurophysiology of the time perception, 13 review articles and 06 experimental papers ones sought to demonstrate the underlying functioning of the timing. This function has participation in cognitive processes and motor control in adaptive responses to the environment. Therefore, the researches point out that timing is represented by different oscillators in order to produce an adequate response to the executive functions and cognition. This fact is possible due to the somatosensory calibration between special receivers that perceive and interact with the internal and external environment, either central or peripheral level. In this way, the time intervals interpretation occurs in two ranges, timing sub seconds and supra-seconds. In general, the time intervals interpretation is performed by diffuse brain areas that act in synchronism to correctly develop the timing judgment, which in PD is deficient.

Eight review articles and 22 experimental papers on dopaminergic dysfunction in the TP. Dopaminergic levels orchestrate the timing judgment as they modulate the internal clock speed. Thus, studies involving genetic polymorphisms in humans and genetically knockout rats, pharmacological models and dopaminergic pathway lesions lead to modulations in the internal clock speed and alter the TP, since alterations in dopaminergic concentrations promote an underestimation or overestimation of the time intervals.

The relation between Levodopa and TP was discussed in 01 review articles and 06 experimental papers. Due to the low dopaminergic levels in PD some classical symptoms of the disease are evident, and levodopa has been a drug widely used as a drug therapy. The results demonstrate that levodopa modulates relation in the TP within of supra seconds. In summary, studies using levodopa injection in rats show a compensatory effect on dopamine levels, thus improving performance on time-lapse tasks.

Twenty-one reviews and 11 experimental papers have demonstrated the relation of memory to PD patients and TP. Memory acts on the time interval coding, and among diseases that affect memory and temporal processing, PD is widely cited. Faced with this, the impairment of frontostriatal circuits are pointed out as the main causes of memory dysfunction. This, due to the dopaminergic depletion that acts in the recruitment and evocation of information pertinent to the time intervals consolidated in the memory. In an experiment involving the time estimation tasks performance and monitoring by functional magnetic resonance imaging (fMRI) in PD patients, demonstrated deficits in the timing tasks performance as well as abnormal neural activations throughout the frontostriatal circuit, cerebellum, frontal and parietal regions during the recruitment of the working memory involved in the timing.

We discuss the motor control and TP in Parkinson’s patients with the inclusion of 12 reviews and 11 experimental papers. TP is embedded in the motor control strategy, as it coordinates the muscles and limbs related to a motor strategy skill. Thus, it is established that changes in patterns and connections in the CNS require proper synchronization of muscle forces and joint activations to generate some desired action. Sensory and motor perceptions are processes integrated with temporal events, mainly in the range of milliseconds-per-minute. Therefore, in PD patients the motor control is impaired due to impairment to the basal ganglia, an important structure in the execution and motor planning, specifically through the cortico-thalamic-basal and cerebellum pathways in the time synchronization.

We discuss four review articles and 08 experimental papers on new time perception studies in PD patients. In this context, we assess what’s new about this paradigm. Since, temporal representations are essential to perform motor tasks, because, in motor planning, the CNS performs an accurate timing. Some studies investigate the involvement of dopamine in the TP through pharmacological models involving the administration of agonist drugs and dopaminergic antagonists in concomitance with motor perception in different sensorial modalities. In principle, Parkinson’s patients underestimate the supra-seconds. Among them we present some of the experimental studies in Table 1.

**Table 1:** Summaries of the main methodologies and their results

Author	Study	Protocol	Results
Silva et al., 2015 [18]	Experimental Study	2 experiments were performed with young, healthy elderly and PD participants. In the 1st experiment, the task consisted of temporal order intervals and in the second experiment, it required precision in the PSE.	In Experiment 1, patients with PD were less accurate than healthy and young adults, while healthy elderly subjects were less precise than young ones. In Experiment 2, PSE was 29 ms for young, 121 ms for healthy elderly and 283 ms for PD patients.
Wiener et al., 2014 [69]	Experimental Study	25 participants underwent fMRI while performing a task of temporal discrimination and color. In addition, the polymorphisms of *DRD2/ANKK1*-Taq1a.SE genes were genotyped.	Better performance on time discrimination versus color was associated with a greater activation in prefrontal and subcortical regions associated with time. A1 carriers of the Taq1a polymorphism showed relatively poorer performance on time discrimination, but not on color. However, in the fMRI greater activation in the striatum and in the right dorsolateral prefrontal cortex and smaller volume in the cerebellum. These results suggest that performance differences in a time discrimination task are attributable to the *DRD2/ANKK1* genotype.
Lake and Meck, 2013 [51]	Experimental Study	22 healthy volunteers were tested in peak interval timing procedures, followed by treatment with d-amphetamine, haloperidol and placebo.	Drug effects were observed, so were encountered two different patterns of timing behavior. In the first standard, d-amphetamines produced shifts to the left in the timing, while haloperidol produced shifts to the right. The second pattern was the opposite of the first pattern.
Balci et al., 2013 [67]	Experimental Study	Participants performed the task of modified interval peak and genotyping of three different gene polymorphisms (*COMT* Val158Met, *DRD2/ANKK1*-Taq1a and *SLC6A3* 3 ‘VNTR).	Participants anticipated the timed response when a higher reward was expected in the absence of changes in decision time or perceived time. The results showed that the reward alters the decision limits rather than the clock speed and that these effects are specific for *COMT* and *DRD2*, which constitute a balanced transmission of prefrontal and striatal dopamine.
Miller et al., 2013 [130]	Experimental Study	28 PD patients performed the finger beats synchronized with tone sequences at time intervals of 500 ms, 1000 ms or 1500 ms, when “ON” for levodopa or the placebo pill.	Patients were less synchronized with the target time interval of 500 ms compared to the larger ones, however, neither the medication status, the affected hand nor the time affected the accuracy and variability of the synchronization.
Wiener et al. 2011 [9]	Experimental Study	65 people performed the temporal discrimination task with intervals of 500 and 2000 ms, spontaneous motor timing task. Genotyping was performed for the *DRD2/ANKK1*-Taq1a, *COMT* Val158Met and *BDNF* Val66Met polymorphisms.	A double dissociation for time discrimination: the *DRD2/ANKK1*-Taq1a (A1+ allele) polymorphism was associated with significantly greater variability over 500 s, while the *COMT* Val158Met (Val/Val homozygotes) polymorphism was significantly associated with greater variability only for the duration of 2000 ms. In addition, the *DRD2/ANKK1*-Taq1a polymorphism was associated with a significantly slower motor timing.
Harrington et al., 2011 [131]	Experimental Study	The subjects composed the control and PD groups on “ON” and “OFF” therapy for DA, under evaluation of fMRI when performing a time perception task.	The results indicated the impaired timing of PD due to nigrostriatal and mesocortical dysfunction in systems that measure timing processes and non-temporal activities. However, perceived deficits in time were not improved by DA treatment, probably due to an inadequate restoration of effective corticostriatal connectivity.
Lewis and Miall. 2009 [102]	Experimental Study	5 participants estimated the time in a range of intervals (68 ms to 16.7 min), where he CV was examined.	Evidence shows a continued logarithmic decrease in CV as the intervals increase. This context, along with other reports demonstrates a scalar property violation in timing data.
Rakitin et al., 2006 [76]	Experimental Study	32 elderly and 32 healthy young people divided into placebo or levodopa groups. They trained to produce two target time slots (6 and 17 s). In addition, participants performed an accelerated reaction time task.	The results indicate that elderly participants show duration-dependent timing errors that are greater than in young people. The Levodopa use produced elongated time perception of both intervals. The aging and effects of levodopa did not interact. And aging retarded RT and increased the variability of RT, but levodopa had no effect on RT.
Lustig and Meck, 2005 [57]	Experimental Study	10 volunteers divided into 2 groups with 5 participants. In the control group, there was no drug exposure. Haloperidol group received doses of haloperidol before and during the experiment. Participants were tested for peak intervals of 7 and 14 s.	There was a deviation to the right at peak time, which increased because of feedback and was reinforced by chronic treatment with haloperidol. These data suggested a shift in the underlying signal duration representation as a function of the feedback spacing that depends on both changes in working memory and the internal clock speed.
Haslinger et al., 2001 [74]	Experimental Study	8 PD patients and 8 healthy participants underwent three fMRI executions while performing movements with joystick in free movement chosen every 7–15 s. The joystick was monitored for the online record of performance parameters, along with pacing tone time and fMRI acquisition parameters.	The control group compared to patients with and without levodopa showed impaired activation related to movement in the supplemental motor area and hyperactivation in the primary motor cortex and the lateral premotor cortex bilaterally. In this context, levodopa improved the motor initiation impairment in the supplemental motor area and decreased the hyperfunction of the lateral premotor and M1 found in Parkinson’s disease.

*SOA* Stimulus onset asynchrony, *fMRI* functional magnetic resonance imaging, *PI* Peak interval, *PSE* point of subjective equality, *DA* Dopamine, *RT* tempo de reação acelerada, *PD* Parkinson’s Disease, *CV* Coeficiente de variação

## Discussion

In the present review, we analyze the literature on the association among TP, cognitive aspects, and PD. We included 144 papers, and the findings show the potential role of dopaminergic modifications as a means, which modulates the levels of neurochemistry in the neural synchronism in timing and cognitive phenotypes in PD.

On the other hand, a wide heterogeneity was documented regarding the adopted techniques and analysis, as well as for sample characteristics and the adverse events that were investigated. In the following paragraphs, we sum up the evidence for the PD association on the TP and cognitive aspects.

### Neurophysiology of time perception

Timing is an innate condition for all species, being an essential component in physiological regulation and adaptation to environmental conditions [22, 23]. Thus, an example of its involvement in cognitive and motor control demand would be the programming to grab an object before it falls, arranging the movement execution in the time interval needed to successfully grasp the object. The cognition in the motor demand promotes the linear timing synchronism, by means of a metrical time interval representation and thus, the occurrence of consecutive events measured in domains of milliseconds to hours [24, 25].

Gupta [26] proposed that the time dimension is represented by different neural oscillators and with regular response to the time interval. Receptors that promote interaction with the external environment also have a fundamental representation for the time interpretation and for motor responses. Such a model was constructed for the processing of short and long intervals, which are of great importance in executive functions and cognition [27]. In addition, different types of neural oscillators composed mainly of sensory neurons and those of excitation/inhibition synchronization interconnect the temporal processing by oscillators embedded in different neural networks, modulated by properties of connections and nature of impulses input (axonal size, nature of the synapses), as well as short duration synaptic plasticity. Accordingly, the process of sensorial feedback and motor changes the rate of changes in the of time codification frequency, determining the temporal passage codification [26].

Moreover, current models of time perception were proposed, among which, the frequency beat model of spiny neurons, aims to indicate mechanisms of a dynamic oscillatory result and characteristics of the neurons involved in each neural circuit [28]. In this model, cortical neurons oscillate temporally in a stable manner, but at different frequencies producing distinct patterns of activity over time [29]. The spiny neurons detect a specific oscillation pattern corresponding to a temporal event, whose action has a single output unit in the same period of time, so that the potentials are accumulated and are grouped by the striatum [30]. Thus, in the cortico-striatal relationship, spiny neurons see a certain pattern among active oscillators coming from dopaminergic neurotransmission, whose different frequencies coincide with specific points of time [31]. Subsequently, learned time interval patterns are strengthened through dopaminergic release, based on the memory of previous experiences with a given time event [30].

Therefore, the coding of time intervals occurs in two domains of time intervals, above and below 1 s [32]. In this context, they determine that for different time domains distinct recruitments brain oscillators [33]. Thus, some encephalic areas are demonstrated as regions activated to perceive time, such as prefrontal cortex, parietal cortex, cerebellum, basal ganglia and primary sensorimotor cortex [34–37]. Accordingly, the most accepted idea for the mechanism by which the CNS performs TP is that diffuse brain areas, in an organized and synchronized way, perform the time intervals interpretation. However, it is noteworthy that in the millisecond range the cerebellum is the most activated region, whereas for supra seconds it involves other regions, such as the basal ganglia [2, 6, 38, 39].

### Dopaminergic dysfunction in the time perception

Dopamine exerts brain regulation in temporal judgment activities [40]. It has been related to executive functions (e.g. motor control, decision making, memory, and attention) [41, 42]. Thus, dopamine exerts modulating activity of the internal clock speed [12, 13], acting as a neurobiological substrate for accumulator-pacemaker pulses according to the Scalar Expectation Theory, in which a series of pulses is produced by an internal pacemaker in the presence of an event; These pulses are collected, counted, and then compared with the stored representations, to allow time-judging [32, 43]. Therefore, changes in dopamine concentration through genetic polymorphisms [44–48], pharmacological models [49–51] and lesions that destroy the dopaminergic pathway [11, 13, 52] promote variations in the internal clock speed and may modify the activities executive performance, perception and judgment of an event [6, 11, 14, 53]. Models proposed by several studies aim to promote the understanding of how the modifications in functional neurobiology approximate or not the risk of developing diseases, including PD, attention deficit hyperactivity disorder, and tendency to drug abuse [48, 54, 55].

Dopaminergic dysfunction in relation to pharmacological models that modify the dopamine system (i.e. cocaine, methamphetamine and haloperidol), are developed by animal testing, in which drugs have injected that increase dopaminergic concentrations such as methamphetamine and cocaine, which promote a left shift in the time judgment, consistent with the time underestimation [11, 49, 51, 56]. In contrast, administration of dopaminergic receptor antagonists such as haloperidol [57] leads to overestimation of time intervals, increasing response time [51, 56]. In relation to dopamine concentration signaling and modulation, German et al. [58] found the main involvement of the dopamine transporter (DAT) and the vesicular monoamine transporter (VMAT) -2 in the transport of presynaptic dopamine to the terminal synaptic vesicles, respectively. Since disturbances in these transporters modify the process of dopaminergic regulation, in which it influences distortions in the time estimation. Consequently, these complexes are determined by means of phosphorylation, protein-protein interactions and changes in intracellular localization [58].

In animals and human experiments, the importance of the prefrontal cortex in the dopaminergic pathway is well established. It acts as a connector with the striated body [59, 60]. Thus, within the striatum, two types of dopamine D1 and D2 receptors are located in striatal interneurons and in the terminal regions of cortico-striatal neurons, respectively. The study proposed by Meck et al. [8] show that the circuits involving the prefrontal cortex and subcortical regions act to synchronize the time interval over a second-to-minute range. Therefore, cortico-subcortical integration plays a key role in the estimation and reproduction behavior of time, which is modulated by dopaminergic concentration. The interactions between dopamine and glutamate transmission in the striatum allow for the clock speed modulation, predominantly by mechanisms mediated by D2 receptors [61–63]. In complementation to the questioning, tests with rats knockout for DAT, it is seen that the genetic changes promote poor performance before the tasks of peak interval, since the deletion prevents the expression of the dopaminergic transporter and increases the extracellular dopamine, as well as decreasing receptors D1 and D2, thus achieving functional loss in the receptors [6, 64].

Accordingly, the loss of D1 and D2 receptors decreases the integrity of striatal neuron populations and thus impairs inaccuracy in timing judgment [13] this decrease is seen in post-mortem brain samples from patients with Alzheimer’s dementia [59]. The expected changes in striatal density decrease are determined by unique nucleotide changes, especially the *DRD2/ANKK1*-TaqIA polymorphism that leads to the change of glutamate by lysine (Glu713Lys) in *ANKK1*, altering the expression of *DRD2*. Therefore, two alleles (A1 and A2) may be identified, since the presence of one or two alleles A1 is associated with the reduction of the D2 receptor in areas of the striatum, with greater observation in the caudate nucleus and putamen [65, 66]. In relation to TP, the neural connections in performance tasks in the time estimation have shown that the participants in the acquisition of judging the interval of time, which have promoted a greater time underestimation [65, 67–69]^.^

### Levodopa and time perception

PD is characterized by dopamine deficiency in the nigrostriatal pathways, and thus levodopa is a drug that helps to treat parkinsonian syndromes in order to improve the clinical picture, increasing the patient life quality. Levodopa is transformed into dopamine by the enzyme dopa-decarboxylase [70]. As dopamine does not cross the blood-brain barrier, therefore its administration is not possible, so levodopa bypassing this barrier may be rapidly decarboxylated and transformed by enzymes into dopamine, hence it becomes the choice of therapeutic drug administration in order to minimize cognitive and motor deficits resulting from low dopaminergic levels [71].

Dopamine depletion in PD patients leads to timing deficits in motor behavior. Generally, after administration of levodopa occur a significant improvement in temporal performance [72]. Although levodopa improves motor function, it has been proposed that it impairs some cognitive aspects such as learning, as it increases the tonic dopamine and obscures phasic changes that are necessary for learning [73]. It is believed that dopaminergic medication ‘normalizes’ the dysfunctional pattern of neuronal activity during simple motor tasks [74, 75].

Levodopa restores temporal performance in rats with CNS lesions [52]. A study relating aging and levodopa found that the administration of levodopa during a time production task elongated the target ranges, that is, under specific conditions, the timing is mediated by dopamine [76]. Bussi et al. [10] observed that daily injections of levodopa improve temporal performance in peak interval processing in rats with circadian disturbances, suggesting that daily dopaminergic increase influences the precise performance in the timing task, as well as the effect of levodopa on the time interval, may mimic the daily increase in dopamine levels in the striatum.

### Time perception and memory in Parkinson’s disease patients

Due to the variability that may occur in clock speed and in the process of storing time memories, the stored value for each external stimulus may be different, despite equivalent target times of reinforcement. Given this, different temporal memories may be selectively coded and retrieved to guide behavior, which means that errors associated with these memory processes may have profound effects on behavior [77]. Thus, because interval time requires executive resources, factors and diseases that promote changes in attention, clock speed, memory or decision-making, affect in different ways the temporal processing [33]. Among diseases that affect memory and temporal processing, PD is constantly reported [78–81].

Although PD is a neurodegenerative disease commonly characterized by its motor characteristics, there is a growing recognition of the impact of the disease on cognition, which influences patients’ quality of life [82]. The most common cognitive deficits in PD are deficits in attention, executive functioning and visuospatial processing, although patients may also present different degrees of memory loss, which may in some cases be more related to recovery than to a problem of intrinsic coding [79, 81–83]. With the development of different therapeutic interventions to minimize the disease motor effects [84], the analysis of neurobiological bases of cognitive dysfunctions in PD has been increasingly sought in order to develop treatments that attenuate the characteristics of dementia associated with disease, including memory problems [85].

Therefore, the breakdown of frontostriatal circuit due to the depletion of dopamine in the basal ganglia and the prefrontal cortex are indicated as the main underlying causes of memory dysfunction even in the early stages of the disease [86]. However, in addition to the search for associating dopaminergic dysfunction with memory dysfunction, explanations have been sought in other domains, which include the influence of cholinergic treatments that offer some clinical benefits [85]. Also, although striatal dysfunction and dopaminergic transmission are pointed to explain TP deficits in the PD individual, problems of working memory and executive functions are also potential causes [64].

Among the main types of memory characterized, working memory is important to actively maintain a certain amount of information to allow its manipulation [87], which is essential during the processing of temporal information [88]. Variation in normal capacity and deficits in working memory may often be explained by the efficacy and integrity of basal ganglia and dopaminergic neurotransmission [89]. This contributes to overcoming the outdated view that the basal ganglia are simply involved in controlling movement, mainly due to the various circuits in this region with cognitive areas of the cerebral cortex [90]. In neuroimaging studies, basal ganglia are activated by the initial storage of temporal information in working memory, while the prefrontal cortex is activated by its subsequent retrieval and comparison with ongoing stimuli [4, 64]. In another experiment, using PD patients as a model for studying the contribution of the frontostriatal circuit to working memory. Uitvlugt et al. [91] applied verbal and spatial memory tests, when tested in the condition without dopaminergic medication, PD patients tended to respond “no” in all tests, supporting that the striatum-pre-frontal pathways influence updates in working memory. On the other hand, PD patients during medication, the intensity of the test did not influence the performance, but rather the presence or absence of distractions, which may be due to the deficiency of the evaluation and selection of stimuli due to dopaminergic overload in the ventral pathway. These results support the striatum-frontal pathway participation for the filtering of spatial or verbal information before entering the working memory system, which is impaired in a PD patient [91].

Leverenz et al. [83] in an experiment with PD patients tested in or out of treatment with levodopa, found impairment in the maintenance, retrieval and manipulation of information from within working memory in patients. However, levodopa medication improved the working memory deficit in patients, but did not improve the deficit in an independent care task, demonstrating that the neuropathology of work memory deficit is related to the exhaustion of dopaminergic transmission [83]. However, it is also evidenced that dopaminergic therapy may contribute to impairment and not improve executive function deficits due to dopamine baseline shift necessary for normal functioning of working memory and executive control [64, 92]. In addition, PD patients have timing deficits that are more consistent on the second scale compared to milliseconds, which may be related to the higher memory load and attention needed for longer intervals, indicating that the dopaminergic loss influences not only direct deficits on the clock speed, but also related to memory and decision processes [93].

Interval time deficits in PD patients are usually interpreted as resulting from deficits in nigrostriatal dopamine depletion affecting circuits involving the basal ganglia [27, 37]. However, it has been shown how the disease may profoundly influence the function of other areas through alterations in the neural networks [94] as well as the atrophy of the gray matter of different areas [95, 96]. In addition to the basal ganglia, dysfunctions during TP tasks involve changes in the executive processing in the frontal cortex [97] which may contribute to TP deficits in PD patients [64], since the medial frontal region and medial pre-motor networks are constantly activated during timing tasks [4, 6]. In one experiment, Harrington et al. [64] applied timing tasks in PD patients, comparing two-time intervals during the collection of fMRI. This study found temporal deficits throughout the frontal and cerebellar networks, and found abnormal activations in the frontal and parietal regions, which are typically associated with executive processes such as working memory.

Studies carried out with rats also suggest that both dopaminergic mesocorticolimbic pathways and cholinergic septo-hippocampal system may functionally interact to regulate various aspects of cognition. In PD patients with dementia, occur an additional loss of cholinergic neurons (Ch4) from the basal ganglia, which present plexus in cortical regions. This demonstrates that in addition to the dopaminergic dysfunction associated with internal clock speed, such as cholinergic dysfunction that is associated with dementia states in patients with different executive functions, including memory related [98]. Thus, it is increasingly believed that dementia in PD is probably secondary to a wider neurodegeneration of dopaminergic transmission [99].

Circadian interruption has negative consequences for the physiological homeostasis throughout the organism [100], including cognitive functions such as memory [101] and perception of short time intervals [102]^.^ Considering that initial theories represented a passive role of sleep to improve memory, current theories highlight an active role for sleep in which memories go through a process of consolidation of newly acquired information [103, 104]. It is also possible to detect memory dysfunctions associated with PD in subjects with Rapid Eye Movement (REM) [105]. Sleep disorder in PD patient with the behavior of sleep disorder has longer disease time, more severe motor and non-motor symptoms and worsening of life quality [106]. In addition, while there are effects on memory and executive function associated with poor sleep in PD, the effects were driven by a small number of studies [107]. The importance of the proper sleep-wake cycle, as it is important for memory and other executive functions, ends up having an influence on TP. In a study observing the electroencephalography during the wake over TP, they observed that the wakefulness level significantly influences the temporal perception of external events [108]. This may be explained by the influence of the circadian system on the interval time during the regulation of dopamine levels in the brain [109]. Therefore, circadian dysfunctions during PD may help to explain the secondary problems involving memory and TP.

### Motor control and time perception in Parkinson’s patients

TP is involved in a variety of executive functions [53], in particular, it has been largely related to the motor control strategy [110] which corresponds to the process in which humans and animals use neural recruitment in order to play and coordinate muscles and limbs Involved in the performance of a motor skill [111]. Thus, because TP is embedded in this process of developing such ability, it needs an integration of sensory information (i.e. information of the environment and information of the physiological constitution itself), in order to promote proper synchronization of muscle forces and joint activations to generate some movement or action [112]. In this way, the pattern changes in the activities and connections of the CNS [113] are established, resulting from the interaction of signals transmitted through the communication linking the internal perception to the external environment [26]. In other words, perception and motor ability are based on signals from the brain, passed through the CNS to converge in the musculature, signals that generate displacements, and the forces needed to adapt to the environment. The alterations resulting from external oscillators act on the generation of sensations that are interpreted by the CNS and subsequently performed, originating from the feedback cycle in sensorimotor integration [114].

Similarly, the understanding of motor and sensory perception in processes integrated with temporal events is arranged in behaviors in the time domain in the range of milliseconds-a-minute, which makes it fascinating, since the basic electrophysiological properties of neurons operate on a scale of milliseconds time [24]. Therefore, motor control and rhythmicity studies are studied in principle, using animal models, which evidences timing circuits by recording neuronal firings, whereas studies in humans are determined by the neuropsychological activities of temporal events, motor or discriminations of time intervals [115]. Thus, through these studies, elucidate how the motor control and TP are deficient in Parkinson’s patients, which have damage in the basal ganglia, which have indicated to be the central structures in the execution and motor planning, as well as actuation of the cortical circuit -thalamic-basal and cerebellum in time synchronization and timing [116]. Taken together, these data suggest that a timing and motor control mechanism associated with the basal ganglia interact with areas dependent on this neural integration (i.e. prefrontal cortex, supplemental motor area, motor cortex, and pre-motor) [117]. Accordingly, the idea of a temporal event synchronization hub to determine the motor rhythmicity, a distributed and interdependent network used to investigate the abstract properties of the reproduction interval adjustment and tasks execution that demand motor behavior.

The basal ganglia and cerebellum are subcortical structures involved in motor planning and cognitive aspects, in addition, the association with the motor cortex allows organizing the processing of information related to motor action [118–120]. In particular, basal ganglia are considered essential to facilitate the desired movements; furthermore, they are enhanced by dopaminergic fibers and thus inhibit undesired movements; while the cerebellum helps fine motor adjustment. In this sense, the synchronization of the basal and cerebellum ganglia is involved in sensory processing and the timing capacity of motor control and learning [121–124]. In view of this, the motor cortex is interconnected with cerebellum and basal ganglia, and others structures [125], which together are responsible for providing flexibility and adjustment over time of reproduction and motor production in tasks that demand repetitive behavior, besides modifying the local architecture in recruiting areas involved in motricity. The representations of movements through these cortical areas have strong interconnections [119, 126], which are very dynamic and with a rapid reorganization in motor time tasks [127].

In principle, PD modify neural morphology, which results in changes in perceptual function, concomitantly influencing various executive functions, especially motor control. Since the neural system functions as an integrated network involving several communication circuits [128]. Above all, PD is characterized by a disordered timing of movements, and these changes manifest in the form of bradykinesia. In this context, bradykinesia is a disturbance of voluntary movements that directly interferes with the daily life of Parkinson’s people, especially in fine motor skills, because it makes planning, execution or initiation of movement difficult [116, 129].

The study by Miller et al. [130] with twenty-eight PD patients in the mild to the moderate stage was performed in order to evaluate the potential of striatal dopaminergic degeneration, as well as its association with the performance of sensory-motor synchronization. Thus, the results suggest that both the basal ganglia and dopamine are inherent in motor timing, regarding the degree of dopaminergic degeneration to explain the differences between parkinsonians in the task of sensory-motor synchronization [130]. Harrington et al. [131] have used functional magnetic resonance imaging to show that cortical systems associated with working memory as well as sensorimotor areas are impaired in PD patients [131], since degenerate dopaminergic pathways between the cortex and basal ganglia contribute to deficiencies in neural synchronization responsible for cognition, emotion, perception and motor function [132], promoting an imbalance of executive functions.

### New studies on time perception and Parkinson’s disease

TP has a subjective and important character for the understanding of reality, as well as performance in executive functions in daily activities [54]. In this context, time representations are essential to perform several motor activities, because when planning a motor act, the CNS performs an accurate adjustment of time and space [133]. Therefore, new studies of TP are feasible in order to understand the distorted behavior in the time intervals interpretation, this influenced by the dopaminergic levels reduction, which interferes in the synchronization and timing judgment [134, 135].

Studies that determine the mechanisms of neural networks that modulate cortical activity over time (i.e. synaptic plasticity, neural adaptation, and neural circuit dynamics) are crucial to understanding the neurofunctional dynamics behind timing in PD patients [20, 136]. In this way, neuronal cells respond to stimuli with different timed responses, that is, the stimuli activate the cells that oscillate at different frequencies and phases [137]. This indicates that the temporal differentiation of the neural firing processes is necessary for several TP models, which depends on the intensity and magnitude of the stimulus [138, 139]. This occurrence has been observed in individuals with PD presenting a reduction in the internal clock speed and consequent underestimation of the time perception, by means of neuropsychological activities, reproduction, estimation and discrimination of the time intervals [140, 141].

The basal ganglia have their function impaired by the alteration of the nigrostriatal pathway that protrudes into the caudate and the putamen. Studies investigate this involvement of dopamine in the perceptual capacity of time through pharmacological models involving the administration of dopaminergic agonists and antagonists in concomitance with motor activities or discrimination of visual and sound stimuli [142, 143]. To date, some neuroimaging studies have investigated the neurophysiological basis of time deficit in PD [48]. However, these studies examined the motor synchronism of relatively short durations, which makes it difficult to differentiate the time relation and motor activity. In contrast, another study examined TP in PD using the comparison interval tasks with durations of 1.2 s and 1.8 s [131], which demonstrates different areas of the brain in association with the deficiency found in PD patients during the coding and timing decision. Striatal dysfunction was found during the two phases, but the working memory capacity composed by the prefrontal dorsolateral cortex (DLPFC), parietal cortex and cerebellum were only disturbed during the coding of the time intervals. Despite all the researches carried out in this field, there are still doubts about the exact nature of TP problems in PD individuals, for instance, Mioni et al. [144] investigated TP in PD, comparing 20 PD participants and 20 control individuals without the disease in explicit (bisection task) and implicit (foreperiod task) timing tasks. The results showed that PD participants presented a preserved ability to perceive the implicit time, but underestimated the explicit one, besides a higher variability of timing when compared to the controls. In this way, they showed a selective fault in the explicit timing.

## Study limitations

The lack of standardization of stimuli and modalities of tasks (sensorial, intensity, size and complexity) in studies involving TP disfavor unanimity in the conclusions. In this context, other factors contribute to this fact, the sample size may impel less precision, for instance in the dopaminergic levels of the patients involved in the study.

## Conclusion

In the present study, cognitive impairments are a hallmark of PD and include disturbances in executive function, working memory, attention, and motor control, all of which are essential for accurate and precise timing. We demonstrate that cognitive processes inbuilt in timing that underlie performance on cognitive tasks and that many are the encephalic areas and functions involved and modulators in the time perception performance. In addition, PD was tested with pharmacological models in comparison to healthy controls for time productions in the supra-seconds range, suggesting that the timing deficits in patients with PD are secondary to the integrity of the basal ganglia, which is crucial for timing in both the sub and supra-second ranges. However, it remains without a definite conclusion about which specific area the timing performs. Based on our results we may affirm that executive functions, such as memory and attention are indispensable constructs for perceiving time, as well dopamine levels are widely reported as neuromodulators of time intervals interpretation and motor timing in adaptive responses to the environment.

Finally, we must not forget the importance of the dopaminergic neurochemical mediation at the neurobiological aspects of TP in all levels: the molecular, neurofunctional; psychopharmacology, and motor control in PD. Studies about TP, which are useful to investigate the neuroanatomical substrates of time interval in PD, providing a functional overview neurochemistry in mechanisms for time synchronization. In summary, the approach of the dopaminergic influence in PD is useful in Neurosciences, since it allows us to help in the elucidation of behavioral phenotypes of patients with PD and study the areas of the brain that are involved in the dopaminergic circuit, and their integration with the perceptual time mechanisms.

## Abbreviations

A1: Alleles
A2: Alleles
Ch4: Cholinergic neurons
CNS: Central nervous system
D1: Dopamine receptors
D2: Dopamine receptors
DAT: Dopamine transporter
DLPFC: Prefrontal dorsolateral cortex
fMRI: Functional magnetic resonance imaging
PD: Parkinson’s disease
REM: Rapid eye movement
TP: Time perception
VMAT: Vesicular monoamine transporter
